# Are HIV, HBV and HCV Voluntary Counseling and Testing Programs Needed in Balkans?

**DOI:** 10.4274/balkanmedj.2017.1229

**Published:** 2018-03-15

**Authors:** Camelia Sultana, Ana-Maria Schweitzer, Mihaela Bogdan, Simona Ruta

**Affiliations:** 1 Carol Davila University of Medicine and Pharmacy, Bucharest, Romania; 2 Stefan S. Nicolau Virology Institute, Bucharest, Romania; 3 Baylor Black Sea Foundation at Centrul Clinic de Excelenta, Spitalul Clinic de Boli Infectioase Constanta-Baylor College of Medicine -Texas Children’s Hospital- Abbott Fund-AbbVie Foundation, Constanta, Romania

To the Editor,

Infections caused due to the HIV, HBV, and HCV are interrelated health conditions with similar behavioral determinants. In particular, Romania is significantly affected by these infections, with the largest pediatric HIV outbreak in Europe being reported during 1985-1992 (1), in addition to the significantly higher rates of chronic viral hepatitis infections than the European average (2). A free, community-run voluntary counseling and testing program was conducted for patients with HIV and viral hepatitis during 2010-2014 in Dobrogea, Romania, a region with high levels of under-diagnosing and under-reporting (3). Informed consent was obtained from all patients, and the study was approved by the institutional IRBs. Serologic screening of 36.132 individuals for HIV, HBV, and HCV infections was carried out using rapid chromatographic tests, and reactive results were confirmed using immunological tests; all subjects filled in a self-reported assessment form regarding behavioral risk factors. Self-referral was the most frequent point of entry into the program (76.9%); people from rural areas were less likely to be referred by a healthcare professional for testing compared with those in urban areas (26.2% vs. 32.6%, p=0.001). During the testing period, the mean prevalence rates of the three tested infections were found to be 0.4% for HIV, 3.2% for HCV, and 4.4% for HBV, with a linear ascending trend identified only for HCV prevalence (Table 1). The highest seroprevalence of HIV infection was observed in young persons (aged 20-39 years), whereas HBV and HCV infections were prevalent among elderly people (aged 50-59 years for HBV and more than 60 years for HCV). Our data suggest a high seroprevalence of unrecognized viral hepatitis infections in older people who were never screened and were exposed to potentially unsafe healthcare-associated parenteral practices before 1990-1995 (4). Although all the tested subjects received a 6-monthly retesting recommendation, retesting was performed for only 24% of the subjects; 0.2% of them were newly diagnosed with HIV (14 patients), 0.3% were diagnosed with HBV (19 patients), and 0.8% were diagnosed with HCV (52 patients). The majority of subjects perceived themselves as having a history of medium- or high-risk exposure (78%), with blood exposure and/or use of unsterilized equipment being more frequently recognized than sexual exposure (69% vs. 26% of the cases, p=0.005). The reported drug use rate was also low (0.08%), which can be expected in a self-referral or a general population screening program (5). Distribution of the self-assessed risks was similar among those tested once and those retested, with a significant association observed between the self-evaluated risk and the retesting probability (p<0.0001) and the strongest association observed with healthcare providers coming back for retesting (p<0.0001). These data suggest that programs aimed at strengthening the implementation of universal precautions in healthcare settings should be a priority. This voluntary counseling and testing program captures a cross-sectional image of health statuses and health-related behaviors of community members and provides important data for stakeholders for the design of a nationwide public health response to HIV and viral hepatitis. In Romania, as in other Balkan countries with a similar epidemiological background, there is a significant community demand for integrated health programs. Implementation of voluntary counseling and testing can complete the overall regional epidemiological picture and concurrently raise awareness about the importance of early detection of infections.

## Figures and Tables

**Table 1:**
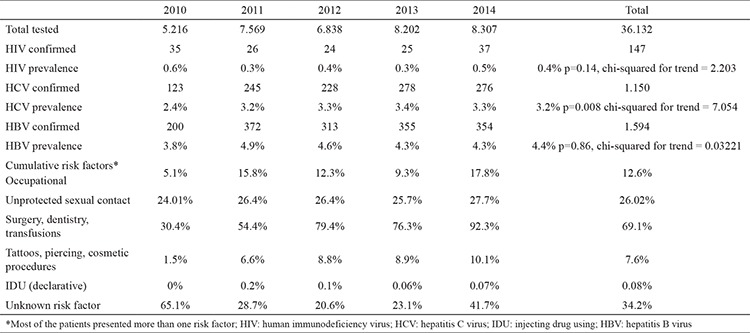
Prevalence of infections and self-declared risk factors during the follow-up period
